# Parent coaching via telerehabilitation for young children with autism spectrum disorder (ASD): study protocol for a randomised controlled trial

**DOI:** 10.1186/s13063-023-07488-6

**Published:** 2023-07-19

**Authors:** Isaac Kwee Mien Sia, Ying Qi Kang, Philina LiXuan Lai, Mythra Mahesh, Shang Chee Chong

**Affiliations:** 1 Child Development Unit, Department of Paediatrics, Khoo Teck Puat-National University Children’s Medical Institute, National University Health System, 1E Kent Ridge Road, NUHS Tower Block Level 12, Singapore, 119228 Singapore; 2 Department of Paediatrics, Yong Loo Lin School of Medicine, National University of Singapore, Singapore, Singapore; 3 Independent Researcher, Singapore, Singapore

**Keywords:** Autism spectrum disorders, Parent coaching, Parent-implemented intervention, Telerehabilitation, Child development, Naturalistic intervention, Routines-based intervention

## Abstract

**Background:**

Early parent-implemented intervention enhances parent-child interaction and improves language skills in children with autism spectrum disorders (ASD). Parent coaching is often delivered as standard care for children with ASD, where parents are taught to apply strategies in their child’s play activities and daily routines to achieve the prior stated goals. However, the ability to conduct parent coaching in physical in-clinic sessions is limited by resource constraints such as clinic space and therapist manpower. Furthermore, parents may experience difficulties with the generalisation of intervention strategies taught in the clinic to their natural home environments. In this study, telerehabilitation is evaluated as an alternative platform to deliver parent coaching for parent-implemented interventions to children with ASD in their homes.

**Methods:**

This parallel-group, randomised, controlled, non-inferiority trial aims to evaluate the effectiveness of parent coaching delivered through video conferencing (telerehabilitation) versus in-clinic (standard care) delivery. Children aged 15 to 48 months (*n* = 200) who meet the cut-off score for ASD on the Autism Diagnostic Observation Schedule-2 are eligible. Parent-child dyads are randomly assigned to receive parent coaching either through weekly telerehabilitation or standard care. The primary outcome is the child’s development as measured by the subscale and composite scores of a standardised developmental assessment. Primary analysis will determine if the lower boundary of the 95% confidence interval for the mean difference in pre-post change between groups exceeds −5 (the non-inferiority margin). Secondary outcomes are the child’s adaptive behaviour, parent-child interaction, parental stress, and family quality of life. Outcomes will be measured pre-intervention, midterm, and post-intervention. Secondary analysis will determine if there is any between-group difference for the pre-post change in scores at the 5% significance level using two-sample* t*-test or Mann-Whitney *U* test.

**Discussion:**

As a randomised controlled trial of a moderately large scale, this study will contribute to the limited existing literature on the effectiveness of parent coaching via telerehabilitation for early parent-implemented intervention for children with ASD. The results of this study will provide insights on whether telerehabilitation is comparable to conventional in-clinic parent coaching in enhancing parent-child interaction and improving language skills.

**Trial registration:**

ClinicalTrials.gov NCT05792449. Registered (retrospectively) on 31 March 2023.

**Supplementary Information:**

The online version contains supplementary material available at 10.1186/s13063-023-07488-6.

## Administrative information

The numbers in curly brackets in this protocol refer to the Standard Protocol Items: Recommendations for Interventional Trials (SPIRIT) checklist item numbers. The order of the items has been modified to group similar items (see https://www.equator-network.org/reporting-guidelines/spirit-2013-statement-defining-standard-protocol-items-for-clinical-trials/).

**Table:** 

**Title {1}**	Parent coaching via telerehabilitation for young children with autism spectrum disorders (ASD): Study protocol for a randomised controlled trial
**Trial registration {2a and 2b}**	ClinicalTrials.gov Registration number: NCT05792449
**Protocol version {3}**	Protocol Version 8; 19 August 2022
**Funding {4}**	Health Services Development Project funded by Ministry of Health, Singapore
**Author details {5a}**	Isaac Sia, Child Development Unit, Department of Paediatrics, Khoo Teck Puat-National University Children's Medical Institute, National University Health System, Singapore, Singapore Kang Ying Qi, Child Development Unit, Department of Paediatrics, Khoo Teck Puat-National University Children's Medical Institute, National University Health System, Singapore, Singapore Philina Lai, Child Development Unit, Department of Paediatrics, Khoo Teck Puat-National University Children's Medical Institute, National University Health System, Singapore, Singapore Mythra Mahesh, Independent Researcher Shang Chee Chong, Child Development Unit, Department of Paediatrics, Khoo Teck Puat-National University Children's Medical Institute, National University Health System, Singapore, Singapore
**Name and contact information for the trial sponsor {5b}**	Michelle Law Michelle_LAW@moh.gov.sg
**Role of sponsor {5c}**	The study sponsor does not have any role in the study design; collection, management, analysis, and interpretation of data; writing of the report; and the decision to submit the report for publication. The ultimate authority over any of these activities reside with the authors.

## Introduction

### Background and rationale {6a}

Increased prevalence of ASD [1] has made innovative and effective intervention programmes, as well as service delivery models, ever needful. For example, early intervention in the form of parent coaching has now been shown to have good outcomes [2] and has become an established evidence-based intervention [3, 4]. However, although parent-implemented intervention is naturalistic—introducing behavioural strategies during daily routines and play activities—many parents need support to generalise strategies taught in clinical settings to home and community settings [5].

Studies evaluating the use of telerehabilitation in autism intervention have largely focused on its feasibility, fidelity, and acceptability [6]. The application of telerehabilitation in parent coaching is relatively better studied [7, 8], exploring the use of online modules [9], demonstration videos [10], or synchronous video conferencing sessions [11]. These studies used single-subject or quasi-experimental designs and randomised trials to demonstrate improvements in both parents’ skills and children’s behaviours. However, there remained a lack of large randomised controlled trials that examined the effectiveness of telerehabilitation in comparison with standard care [12].

In Singapore, ASD is the greatest contributor to disease burden for children 0 to 14 years [13] and affects nearly 1% of the population [14]. The Child Development Unit (CDU) at the National University Hospital is one of two nationally designated sites offering multidisciplinary outpatient services for children below the age of 7 diagnosed with ASD or other developmental, learning, and behavioural difficulties. Children with ASD are enrolled into the Foundational Skills Program which is adapted and developed from Cumine et al. [15], where therapists coach parents to implement early intervention strategies across four core domains—social interaction, social communication, play skills, and participation in daily routines (i.e. the Foundational Skills Curriculum, FSC).

The CDU faces several common but critical challenges in meeting the needs of children with ASD. Firstly, a heavy caseload due to the large number of children diagnosed [16] means most experience a long wait for intervention to begin and/or a low frequency of contact with their therapists. Space and room constraints also limit the number of children that can be seen in-clinic. Furthermore, even though the Foundational Skills Program is naturalistic in its approach, parents may find it difficult to generalise what they learn in-clinic to their own home and community settings. For some parents, it is effortful to bring children to clinic for repeated appointments due to child’s behavioural difficulties as well as parent’s busy work schedules.

The telerehabilitation solution in this trial uses video conferencing to address these challenges. Telerehabilitation sessions were shorter than in-clinic sessions as it was anticipated that parent-child dyads in their own homes require less time to transition into and out of therapy sessions as compared to sessions in a less familiar clinic environment. This enables therapists to schedule more sessions and improve access for more patients during their workday. Less clinic space is required to conduct telerehabilitation sessions than in-clinic sessions, allowing more concurrent sessions to take place within the clinic. Finally, through remote real-time coaching, therapists can teach, guide, and empower parents to implement intervention strategies in their home environment immediately.

### Objectives {7}

This study aims to evaluate the effectiveness of parent coaching delivered through telerehabilitation (intervention arm) with standard in-clinic parent coaching (standard arm) for children with ASD. We hypothesise that telerehabilitation will be non-inferior to in-clinic coaching on the primary outcome of children’s developmental skills. Additionally, we hypothesise that telerehabilitation will be non-inferior to in-clinic coaching on the secondary outcomes of children’s adaptive behaviour, parent-child interaction quality, parental stress, and family quality of life.

### Trial design {8}

This is a randomised, controlled, parallel group, non-inferiority trial. Block randomisation will be used with a 1:1 allocation with 100 participants in each arm.

## Methods: participants, interventions and outcomes

### Study setting {9}

This is a single-site study at Singapore’s National University Hospital CDU. The CDU operates family-centred, community-based clinics offering a multidisciplinary service through a team of developmental-behavioural paediatricians, psychologists, occupational therapists, speech therapists, physiotherapists, learning support educators, nurses, and social workers. The CDU assesses and manages children from birth to 7 years old (i.e. Singapore education levels up to K2) with learning, behavioural, and developmental needs. These conditions seen at the CDU include ASD, attention deficit hyperactivity disorder, speech and language disorders, hearing impairments, developmental delays, specific learning disabilities (i.e. dyslexia, developmental coordination disorder, specific language impairment), behavioural difficulties, and children with chronic illnesses at risk of developmental delays. Children with ASD make up 30–35% of the annual case-load of new visits to the CDU.

### Eligibility criteria {10}

Inclusion criteria:

1. Children aged 15 to 48 months
2. Children meet the cut-off score for ASD on Autism Diagnostic Observation Schedule-2 (ADOS)
3. Parent(s) is/are willing and able to give informed consent
4. Families with at least one parent who is digitally literate with the home use of the Internet and access to Wi-Fi
5. Same parent(s) or caregiver(s) (e.g. grandparent) will be in attendance for most interventions as well as review sessions for outcome measurement

Exclusion criteria:

1. No access to the Internet
2. Had previously received or is receiving early intervention for ASD from another provider
3. Genetic disorder, auditory or visual impairment, seizure disorders

### Who will take informed consent? {26a}

Parent(s) or caregiver(s) will be given verbal explanation of the study and a written consent form will be provided. They will be given ample opportunities and time to ask any questions, discuss with their family and friends, and consider options before making a decision. Parent(s) or caregiver(s) will be assured that their care will not be affected whether or not they participate in the study. It will also be emphasised that participation in this study is voluntary and they may withdraw at any time. Written informed consent will be obtained before proceeding with any study procedures. The principal investigator or co-investigators will obtain informed consent in the presence of an independent witness. Assent from participating children will not be sought since they are only 15 to 48 months old.

### Additional consent provisions for collection and use of participant data and biological specimens {26b}

Not applicable; no ancillary studies will be conducted.

### Interventions

#### Explanation for the choice of comparators {6b}

Parent training for parent-implemented intervention strategies for young children with ASD is effective [17] and has been shown to improve social skills, communication and adaptive behaviour in children [18–20], parent skills in implementing intervention strategies, and quality of parent-child engagement [21]. The standard care treatment for children diagnosed with ASD in the CDU is parent-training based on the FSC.

The control intervention in this trial is standard care in the CDU where parent training takes place through in-clinic sessions. Therapists demonstrate implementation of strategies and have parents practise with their children immediately during in-clinic sessions. Therapists also provide parents with clear and detailed instructions and guidance about practising strategies taught in real-life interactions with their children for generalisation of skills. However, therapists are unable to observe children’s behaviours and parents’ implementations of strategies at home directly. Neither can therapists provide immediate, meaningful, and relevant feedback to parents about their performance at home. Parents trained would then be able to practise the skills learned with their children across a variety of settings.

The experimental intervention in this trial is parent training via telerehabilitation. Parents who have experienced early intervention through telerehabilitation have reported that remote observation and coaching while they embed intervention strategies into daily activities facilitates practising of strategies throughout the day [22]. Remote parent training may also be associated with improvement in child outcomes [23], but its effectiveness versus standard care for young children with ASD has yet to be compared.

#### Intervention description {11a}

The FSC focuses on the core domains of social interaction (spontaneous use of gaze, spontaneous maintenance of proximity, imitation, turn taking, initiating, emotional expression and understanding, development of self), social communication (understanding simple verbal and non-verbal approaches, strategies for meeting needs, e.g. requesting, engaging in social interaction, joint attention), play (manipulative/exploratory, structured/constructional, cause and effect/means to an end, interactive/social, pretend play–functional, symbolic, fantasy, social), and participation in daily routines. Parents will be trained in evidence-based approaches such as following their child’s lead, imitation, and modelling, communicative temptations, and playful obstructions during playtime and daily routines. They will be encouraged to interact with their child using strategies that aim to increase their child’s attention and motivation, participation in turn-taking routines, and initiation and response to joint attention.

The parent coaching approach gradually transits from introducing concept and strategies to demonstration then to guided parent practice. Parent-child dyads in the standard arm will undergo a parent coaching programme based on the FSC in-clinic. Participants in the intervention arm will undergo the same programme via telerehabilitation. Parent coaching will be delivered by therapists (licensed psychologists and speech therapists) trained in the FSC and parent coaching. Therapists delivering parent coaching to each parent-child dyad will remain unchanged throughout the study.

##### Programme schedule

The standard arm consists of 16 in-clinic intervention sessions of 60 min (total intervention time = 960 min). The intervention arm starts with two in-clinic intervention sessions of 60 min, followed by 16 telerehabilitation sessions of 45 min (total intervention time = 840 min). Additional time is allocated for in-clinic sessions in order for parent-child dyads to adjust and settle into the unfamiliar therapy environment. This allocation is not given for telerehabilitation sessions in which parent-child dyads receive therapy in their home environments.

For in-clinic sessions, the therapist will coach parents to implement intervention strategies through play activities and help parents identify contexts and activities at home where parents could practise with their children. For the telerehabilitation intervention arm, their first two in-clinic sessions provide the opportunity to explore various intervention strategies with direct demonstration and guidance from the therapist that will serve as a foundation for the subsequent coaching through telerehabilitation. During subsequent telerehabilitation sessions, the therapist and parent will log onto the video conferencing platform (Zoom) and the therapist will be able to observe, look, and listen to the dynamics of parent-child interactions during a given daily routine or play activity and coach the parent remotely in real time.

Both programmes have intervention sessions organised into intervention blocks with breaks in between to provide opportunities for parents to consolidate their learning and to practise strategies taught at home. At the end of each intervention block, the therapist and parents craft goals and identify home routines and strategies that can be used to achieve these goals during these consolidation breaks. Handouts describing the strategies and examples of embedding the strategies within routines will be given to parents for their reference.

##### Session format

Sessions in both programmes will be structured according to the collaborative coaching protocol [24] beginning with checking in, followed by reviewing, observing, reflecting, explaining and coaching of new foundational skills and strategies, setting goals and follow-up planning in the home context, then closing. Each session begins with a 5- to 10-min review and discussion of parents’ progress from the previous session and any other updates. This is followed by a 10-min parent-child play activity. The activity will allow the therapist to observe the parent’s competency in carrying out interventions taught in previous sessions. The therapist will further coach parents in order to strengthen parents’ confidence and competency in using strategies to facilitate the attainment of specific goals in the intervention plan. During the final 10 to 15 min of each session, the therapist will check parent’s clarity, comfort, and confidence in implementing the follow-up plan and provide further input and information as required. The therapist will also address any challenges faced or anticipated in the context of implementing the follow-up plan at home. The therapist will guide parents to implement the follow-up plan using at least two play or daily routines (naturally occurring opportunities) identified by the parent. Adult learning principles will be adopted by the therapist to facilitate active participation in planning and implementation [25].

#### Criteria for discontinuing or modifying allocated interventions {11b}

Participation in the study is voluntary and participants may request to withdraw at any point of the study without penalty. No harm is anticipated for telerehabilitation and/or parent-mediated naturalistic intervention. If the participant is enrolled in Early Intervention Program for Infants and Children (EIPIC) or private early intervention services, they will continue to receive intervention per the study protocol for 10 weeks (child is being assessed in the first 10 weeks of enrolment in EIPIC) or as long as they have not started receiving intervention in the new centre.

#### Strategies to improve adherence to interventions {11c}

The study team will monitor participants’ progress in the study closely to ensure that they attend intervention sessions according to the respective programme’s schedule and timeline. While set timeframes for assessments, intervention, and reviews are important, this study is implemented under operational conditions of a clinical service where scheduling challenges are an unavoidable field condition. A degree of scheduling flexibility is hence built into the schedule of both arms. For parents who miss their scheduled sessions for reasons such as the child or parent feeling unwell or parents’ work-related commitments, they will be contacted to schedule make-up sessions. Buffer periods are incorporated into the programme schedule as breaks in between intervention blocks to allow for rescheduled make-up sessions. In general, where delays from scheduling occur, each child will move to the next phase of the study (i.e. next intervention block) only after they have completed all planned sessions in the previous phase.

#### Relevant concomitant care permitted or prohibited during the trial {11d}

Participants who have been enrolled into the study are prohibited from receiving concomitant early intervention that focuses on similar core domains of social interaction, social communication, play skills, and participation in daily routines from another provider apart from the CDU.

#### Provisions for post-trial care {30}

Participants are not expected to suffer from any harm from participating in this trial. Nevertheless, the National University Hospital will cover medical expenses for study-related injury if any does occur.

### Outcomes {12}

The primary outcome is the mean change of children’s developmental skills from baseline to programme conclusion. Children’s developmental skills such as visual reception, fine motor, expressive language, and receptive language are commonly assessed to determine the effectiveness of ASD intervention [26, 27].

The first of the secondary outcomes is mean change of adaptive behaviour from baseline to programme conclusion. Adaptive behaviour used by children to cope with challenges of daily living is a key distal measure of intervention effectiveness [20, 28].

Joint engagement is a prerequisite skill for social interaction and language development and is often an important component of early intervention programmes [29]. Joint engagement assessment will be completed at baseline, midpoint, and programme conclusion and mean change will be compared.

Equipping parents with strategies to manage and support their child’s behaviour may improve parental self-efficacy and lower relative stress in a parent-child relationship [30]. Change in mean parenting stress from baseline to programme conclusion will be compared.

Placing emphasis on family quality of life guides programmes to make the assessment of families’ strengths and needs a priority and enable families to become the focus of decision. Mean change of family quality of life from baseline to programme conclusion will be compared.

Additionally, to obtain specific information about the effectiveness of parent coaching via telerehabilitation, parents’ fidelity in implementing intervention strategies taught will be assessed at baseline and programme conclusion and compared for participants in the telerehabilitation arm only. Non-healthcare direct and indirect costs of attending intervention sessions will be taken as the mean of costs reported by families across baseline, midterm, and programme conclusion. Parent satisfaction will be assessed at the programme conclusion.

### Participant timeline (Table 1) {13}

**Table 1:** Participant timeline

	**Eligibility**	**Enrolment**	**Post-allocation**			**Close-out**
**Timepoint**	***−t***_***1***_	***t***_***0***_	***t***_***1***_ Baseline	***t***_***2***_ RV A	***t***_***3***_ RV B	***t***_***4***_ Final
**Enrolment**						
Eligibility screen by developmental paediatrician	X					
Informed consent	X					
Eligibility assessment: ADOS	X					
Random assignment		X				
**Interventions**						
Standard			16 in-clinic sessions			
Telerehabilitation			2 in-clinic sessions followed by 16 video conferencing sessions			
**Assessments**						
MSEL	X					X
VABS-III	X					X
PSI-SF	X					X
FSC			X	X	X	X
JERI			X	X	X	X
NDBI-Fi^a^			X	X	X	X
FEIQoL			X			X
Demographic survey			X			
Cost survey			X	X	X	X
Parent satisfaction survey						X

^a^Only participants in the telerehabilitation arm are rated on NDBI-Fi

#### Eligibility screen (-t_1_)

Children under clinical suspicion for ASD according to Diagnostic Statistical Manual of Mental Disorders, 5th edition, will undergo further assessments to determine their eligibility for the study.

#### Informed consent (-t_1_)

Informed consent will be obtained from participants before they undergo any study procedures.

#### Eligibility assessment (-t_1_)

As part of the eligibility assessment, participants will be assessed on ADOS. Those who meet the criteria for autism on ADOS and do not have any of the conditions listed on the exclusion criteria will be enrolled into the study. Besides the ADOS, participants will complete assessments on the Mullen’s Scale of Early Learning (MSEL), Vineland Adaptive Behaviour Scales (VABS-III), and Parenting Stress Index-Short Form (PSI-SF).

#### Random assignment (t_0_)

Computer-generated block randomisation will be used to randomly assign participants to one of the intervention arms—standard or telerehabilitation.

#### Baseline assessment (t_1_)

The therapist will establish a baseline in four core domains—Social Interaction, Social Communication, Play Skills, and Daily Routines. Parent-child interaction based on 4 interactive scenes in the Communication Play Protocol [31] will be recorded and evaluated offline using the Joint Engagement Rating Inventory (JERI). Parents will complete a demographic survey, cost survey, and Families in Early Intervention Quality of life (FEIQoL) survey. For the telerehabilitation group, an additional review will be conducted remotely through telerehabilitation. Parent-child interaction during free play in the natural home environment will be recorded for offline rating using NDBI-Fi.

#### Intervention programme

The standard programme consists of 16 in-clinic intervention sessions while the telerehabilitation programme consists of 2 in-clinic intervention sessions followed by 16 video conferencing-based sessions. The approximate duration of the intervention programme would be 40 to 60 weeks, inclusive of midterm reviews and the final review.

#### Midterm reviews (RV A and RV B) (t_2_ and t_3_)

There will be 2 midterm reviews with the paediatrician and therapist. The paediatrician will review the child’s general developmental profile and progress as well as check in on the family’s needs. The therapist will review the child’s developmental profile on the FSC. Parent-child interaction based on four interactive scenes in the Communication Play Protocol [31] will be recorded and evaluated offline using JERI. A cost survey will also be given to parents. For the telerehabilitation group, two additional reviews coinciding with the midterm reviews will be conducted remotely through telerehabilitation. Parent-child interaction during free play in the natural home environment will be recorded for offline rating using NDBI-Fi.

#### Final review (t_4_)

The child will be evaluated on MSEL. The PSI-SF, VABS-III, cost survey, FEIQoL survey, and parent satisfaction survey will be administered to parents. Parent-child interaction based on four interactive scenes in the Communication Play Protocol [31] will be recorded and evaluated offline using JERI. For the telerehabilitation group, an additional review will be conducted remotely through telerehabilitation. Parent-child interaction during free play in the natural home environment will be recorded for offline rating using NDBI-Fi. The paediatrician will share the results of the evaluations, discuss the child’s progress, and look into any further needs of the family.

### Sample size {14}

The sample size calculation is based on a one-sided non-inferiority test at 95% confidence. We defined our non-inferiority margin as 5 points on the primary outcome measure of MSEL ELC that has a standard deviation of 15 points [32]. The non-inferiority margin of 5 was informed by clinical expertise and existing literature [33]. If there is no truly no difference in the pre-post change between the standard arm and telerehabilitation arm, then 132 participants are required to be 80% sure that the lower limit of a one-sided 95% confidence interval for the difference will be above the non-inferiority limit of −5. We decided to recruit 200 subjects in total (100 in each arm) after considering withdrawals and lost to follow-up. The computation was performed using a proprietary software programmed in R.

### Recruitment {15}

As one of two nationally designated sites offering multidisciplinary outpatient services for children with developmental, learning, and behavioural difficulties, CDU receives a large number of referrals each year. All children who are found to be at risk for ASD by the developmental paediatricians, and who meet the inclusion criteria without any of the exclusion criteria, will be asked if they would be interested to participate in the study. The recruitment process started on 1 January 2019 and was completed on 14 April 2022.

### Assignment of interventions: allocation

#### Sequence generation {16a}

Computer-generated block randomisation is used to randomly assign participants to either the standard or telerehabilitation arm with a 1:1 allocation. The total sample size of 200 is divided into blocks of four with random permutations of two standards and two telerehabilitations allocated in each block.

#### Concealment mechanism {16b}

Randomisation lists in blocks of four are placed into opaque, sealed envelopes and stored in a dedicated locked cabinet. The study team will not know in advance which subject will receive which intervention beyond each block of four as envelopes are only opened as and when participant assignment is needed.

#### Implementation {16c}

Randomisation lists are generated by the study statistician. The assigned study team member will enrol participants if they meet the inclusion criteria without any of the conditions listed in the exclusion criteria. Upon enrolment, the participant will be assigned the intervention arm allocated to their subject number in the order that they are enrolled.

### Assignment of interventions: blinding

#### Who will be blinded {17a}

Raters for the JERI and NDBI-Fi and assessors of the primary outcome using the MSEL at programme conclusion are blinded to participant’s intervention arm. Blinding of parent-child dyads and therapists delivering intervention are not possible.

#### Procedure for unblinding if needed {17b}

No unblinding procedure is set up for this trial.

### Data collection and management

#### Plans for assessment and collection of outcomes {18a}

Developmental skills will be assessed using MSEL. The MSEL is a standardised developmental assessment that is widely used to assess the developmental skills of children from birth to 68 months [32]. The MSEL evaluates motor and cognitive functions on 5 subscales: gross motor, visual reception, fine motor, expressive language, and receptive language. For each subscale, the assessment derives a *T*-score (mean of 50 and standard deviation of 10), a percentile score, and an age equivalent indicating the developmental age the child is performing at. ELC score is derived from four of these subscale scores (excluding gross motor) and will be used to compare the effectiveness of the programme [34]. The ELC has a mean of 100 with standard deviation of 15. Individual subscale scores will be analysed as additional outcomes to examine the impact of intervention on specific functions of the child and for separate assessment of verbal and non-verbal abilities [35]. Test-retest reliability coefficients ranged between .71 and .85 for MSEL subscales [32]. MSEL assessment will be conducted at baseline and at programme conclusion for participants in both groups. Assessors at programme conclusion will be blinded to intervention allocation. Mean change of ELC score from baseline to programme conclusion will be compared across groups. A conclusion that parent training delivered via telerehabilitation is as effective as standard care in improving the developmental skills of children with ASD is reached if the lower bound of the confidence interval of the difference in ELC between groups falls within the non-inferiority margin of -5.

VABS-III is a standardised assessment that utilises a semi-structured interview with caregivers to measure the adaptive behaviour used by children to cope with challenges of daily living [36]. The assessment can be used for children from birth to 18 years, 11 months with or without developmental delays, and covers the main domains of communication, daily living skills, and socialisation. All main domains come with standard scores with a mean of 100 and a standard deviation of 15 and percentile scores. Scores on the three main domains are combined to obtain an overall Adaptive Behavior Composite with a mean of 100 and a standard deviation of 15. All test-retest reliability coefficients were found to be above .60 for all age groups above 3 months [36]. VABS-III assessment will be conducted at baseline and at programme conclusion and mean change from baseline will be compared across groups.

JERI measures joint engagement, communication dynamics, and shared topics during parent-child interactions [37]. Raters blinded to timepoint and group assignment will view video recordings of parent-child interaction and assess the dyad on 8 items of joint engagement using a 7-point Likert scale (see Additional file 1). Raters will be trained based on the training and reliability procedures written by the original authors [38]. The trainer will provide 5 assignments for the trainee to rate independently. The goal is for the trainee to be in agreement (within 1 point) with the established ratings a minimum of 80% of the time. If the trainee is unable to meet this goal, another 5 assignments will be given. Once the raters have been launched to rate the videos in the study corpus, 15% of all interactions will be rated by two raters. The interactions used for the reliability assessment will be randomly selected and the raters will not be aware of which videos are being used to assess reliability. Reliability will be assessed using weighted Kappa [39]. 1-point disagreements will be regarded as agreements in the weights matrix, while greater disagreements beyond 1-point will be graded proportionally. The goal for inter-rater reliability is to never fall below 80%. JERI assessment will be completed at baseline, midpoint, and programme conclusion and mean change will be compared across groups.

Equipping parents with strategies to manage and support their child’s behaviour may improve parental self-efficacy and lower stress. PSI-SF is a 36-item self-report questionnaire assessing the level of relative stress in a parent-child relationship [40]. The questionnaire consists of three subscales: parental distress, parent-child dysfunctional interaction, and difficult child. Subscale scores range from 12 to 60 and the Total Stress score ranges from 36 to 180 with higher scores indicating greater stress. The PD-SF subscale evaluates the parent’s experienced distress in being a parent, the PCDI-SF subscale evaluates the parent’s perception of the extent to which (or whether) their satisfaction or expectations in parenting are met, and the DC-SF subscale evaluates the degree of the parent’s distress resulting from the child’s difficult behaviours. Parenting stress is distress or discomfort caused by childrearing demands that parents experience or by the parents’ inability to meet these demands and manage parenting stressors through regular family-coping strategies. Internal consistency of the domains ranged from .80 to .91 in the original study [40]. Change in raw scores on the PSI-SF from baseline to programme conclusion will be compared across groups.

Improving family quality of life is important for early intervention programmes. This study assesses the family quality of life using the FEIQoL scale [41–44]. The FEIQoL assesses the quality of life across 4 domains: (a) family relationships (problem solving, communication, parenting, relationships with extended family, and family participation in social activities); (b) access to information and services (knowledge of their child's disability, child development, managing challenging behaviours and resources such as support services, medical assistance, and organisations in their community); (c) child functioning (child’s engagement, independence, and social relationships within family daily routines); and (d) overall life situation (fulfilment of family needs in health, financial resources, and employment). An internal consistency of *α* = .94 was established [45]. Family quality of life is measured by the four mean ratings for items in each respective domain. The mean change of ratings from baseline to programme conclusion for each domain will be compared across groups.

NDBI-Fi is an 8-item observational rating scheme that evaluates caregiver implementation of specific strategies that have been identified to be common among naturalistic developmental behavioural intervention programmes [46]. The intraclass correlation for the overall scale was found to be 0.80 in the original study and when compared with fidelity scales of other NDBIs, a positive correlation (*r* = 0.60) was found, demonstrating strong construct validity for the scale [46]. Raters blinded to timepoint will view recordings of parent-child interaction during free play and evaluate parents’ implementation of strategies using a 5-point Likert scale (see Additional file 2). The trainer will provide 6 assignments for the trainee to rate independently. Trainees are expected to obtain reliability, defined as (a) no items are more than 2 points apart, (b) at least 7 items are within a single point, and (c) average ratings are within half a point. If the trainee is unable to meet this goal, another 6 assignments will be given. Once the raters have been launched to rate the videos in the study corpus, 15% of all interactions will be rated by two raters. The interactions used for the reliability assessment will be randomly selected and the raters will not be aware of which videos are being used to assess reliability. Reliability will be assessed using weighted Kappa [38]. 1-point disagreements will be regarded as agreements in the weights matrix, while greater disagreements beyond 1-point will be graded proportionally. The goal for inter-rater reliability is to never fall below 80%. Change on the NDBI-Fi will be calculated by comparing changes on individual rating items and total average, from baseline to programme conclusion, for participants in the telerehabilitation arm only.

The Cost Survey (see Additional files 3 and 4) will measure direct and indirect non-healthcare costs of attending intervention sessions, including transportation costs, additional assistance costs, and productivity losses. These costs will be reported by parents in both groups at baseline, midterm, and final reviews.

The 13-item Parent Satisfaction Survey (see Additional file 5) uses a 5-point Likert scale to obtain parents’ feedback at the end of the programme. Each item requires the parent/caregiver to rate the degree to which s/he agrees with a statement on the scale. Parents will be given this survey at the end of the study and the responses will be compared between the two intervention groups to see if telerehabilitation is as acceptable as standard care.

#### Plans to promote participant retention and complete follow-up {18b}

Participants are given breaks between each intervention block to promote participant retention over the course of the extended therapy programme. Accommodation will be made to participants’ availability as much as possible when scheduling visits without deviating from protocol.

CDU provides transitional care for children pending enrolment into longer-term community-based early intervention services. Participants who have secured a place in such services will continue to receive intervention per protocol until they start receiving intervention from the new provider (e.g. while undergoing the 2.5-month-long entry assessments at the receiving service providers). Study participants who have transited to other early intervention services will also be assessed per protocol at midterm and final review timepoints.

Participants who complete at least one block of intervention (6 sessions) but request to withdraw afterwards will be offered an option to return for an exit assessment before withdrawing. The child will be assessed on the MSEL, while parents will complete the VABS-III, PSI-SF, FEIQoL, Parent Satisfaction Survey, and Cost Survey. A video-recording of parent-child interaction during four Communication Play Protocol scenes will also be recorded for JERI rating. In addition, parent-child interaction during free play will be recorded through video-conferencing in telerehabilitation arm participants for NDBI-Fi rating.

#### Data management {19}

Study data will be collected using paper forms and then entered and managed using REDCap hosted by the National Healthcare Group [47, 48]. REDCap (Research Electronic Data Capture) is a secure, web-based software platform designed to support data capture for research studies, providing (1) an intuitive interface for validated data capture; (2) audit trails for tracking data manipulation and export procedures; (3) automated export procedures for seamless data downloads to common statistical packages; and (4) procedures for data integration and interoperability with external sources. Data integrity checks will be conducted periodically every 6 to 12 months by the principal investigator. All paper forms will be kept under lock and key after results have been entered into REDCap.

#### Confidentiality {27}

Participants were informed during the informed consent process that their participation in the study will involve the collection of identifiable, personal information that will be kept confidential. National University Hospital, National Healthcare Group Domain Specific Review Board, and Ministry of Health will be granted direct access to participants’ original medical records to check study procedures and data, without making any information public.

Screening and enrolment numbers will be assigned and used on all participant assessment forms and analyses. Screening and enrolment number assignments will be recorded and stored separately from study records under lock and key.

Data collected will be stored for 6 years upon study completion per institutional policy. Hard copies will be kept under lock and key while soft copies will be stored on REDCap with restricted access. Thereafter, hard copies will be shredded and disposed of securely while soft copies will be permanently deleted according to institutional protocols and requirements.

Participants will only be referred to by their enrolment number (where appropriate) in any publications resulting from this study.

#### Plans for collection, laboratory evaluation and storage of biological specimens for genetic or molecular analysis in this trial/future use {33}

This study does not involve the collection of biological specimens.

### Statistical methods

#### Statistical methods for primary and secondary outcomes {20a}

Two-sample* t*-test or Mann-Whitney *U* test (depending on the distribution of data points and total sample size) will be used to compare the pre-post change between groups in the MSEL score (primary outcome). Non-inferiority of telerehabilitation to standard care is established if the mean difference between groups is less than 5. Pre-post changes in secondary outcomes as measured by VABS-III, JERI, FEIQoL, and PSI-SF will be compared using either two-sample *t*-test or Mann-Whitney *U* test. Linear regression will be used to adjust for any covariates. Paired-sample *t*-test will be used to compare the pre-post changes in NDBI-Fi in the telerehabilitation arm.

#### Interim analyses {21b}

Interim analyses will be conducted every 6 months by an independent study statistician. The statistician will report the results to the Principal Investigator and the study sponsor (Singapore Ministry of Health). All data available at the time of analyses will be included. All demographic, clinical, and cost data will be analysed descriptively. The trial will be terminated if the research question has been answered adequately according to the statistical parameters set out a priori.

#### Methods for additional analyses (e.g. subgroup analyses) {20b}

A sensitivity analysis excluding participants who transited to community-based early intervention programmes before study completion may be conducted to examine its impact on trial results.

#### Methods in analysis to handle protocol non-adherence and any statistical methods to handle missing data {20c}

Subjects with major deviation from study protocol will be excluded from analysis of the primary outcome which will be carried out per protocol. Certain protocol deviations are expected to occur as this study is being conducted as part of routine clinical care and operations. For example, rescheduling or defaulting of appointments (e.g. due to work commitments, therapist/child’s illness, etc.) are inevitable. Only participants with a major protocol deviation, i.e. not attending at least 80% of therapy sessions, will be excluded from analysis. Missing data due to participant withdrawal will be reported along with withdrawal reasons which will be evaluated qualitatively.

#### Plans to give access to the full protocol, participant-level data and statistical code {31c}

The datasets analysed during the current study and statistical code are available from the corresponding author on reasonable request, as is the full protocol.

### Oversight and monitoring

#### Composition of the coordinating centre and trial steering committee {5d}

The trial coordinating and steering committee will comprise the principal investigator, physician representatives, therapist representatives, and a research assistant. The committee meets monthly initially to address implementation and operational issues and propose adjustments to the study protocol as appropriate. Subsequently, the committee will review and discuss study progress and interim results, as well as respond to any new issues arising or brought up by the representatives over the course of the trial.

#### Composition of the data monitoring committee, its role and reporting structure {21a}

This study does not have a data monitoring committee as the study team considers the intervention being investigated (telerehabilitation) to be safe.

#### Adverse event reporting and harms {22}

Any adverse events and unintended harmful effects of the intervention will be brought up to the attention of the principal investigator immediately for timely response and reporting to the institutional review board as stipulated by regulations.

#### Frequency and plans for auditing trial conduct {23}

Both the National University Health System Research Office and the National Healthcare Group Research Quality Management Unit hold the authority to perform audits on trial conduct. There is no set frequency for audits and study team must ensure readiness for such audits at all times.

#### Plans for communicating important protocol amendments to relevant parties (e.g. trial participants, ethical committees) {25}

Protocol amendments will be submitted to the institutional review board for all modifications that impact conduct of study, potential benefits to participants, or participant safety. These include changes in study objectives, design, population, sample size, procedures, and significant administrative or operational processes. Changes will only be implemented with prior written approval from the institutional review board. If trial participants are affected by the change, they will be informed and asked to re-consent before continuing in the study.

#### Dissemination plans {31a}

Final analysis of the data will be conducted after the end of the study. The findings will be disseminated through abstracts, presentations, and publications which can be accessed by healthcare professionals or the public, as approved by the Principal Investigator.

## Discussion

This paper presents the study protocol for a telerehabilitation study that aims to evaluate the effectiveness of video conferencing-based intervention in comparison to standard in-clinic intervention for young children with ASD. As a randomised controlled trial of a relatively larger scale as compared to other studies, the present study will contribute to the limited existing literature on the effectiveness of telerehabilitation as an alternative service delivery platform for parent training. Another of the trial’s strengths would be the selection of outcome measures which comprise a mixture of children’s skills-based assessments, parent-based questionnaires, and video-based ratings which are able to objectively capture pre-post intervention change in the widest possible way within the child’s development as well as social and family contexts.

The COVID-19 pandemic has fast-tracked the implementation of telerehabilitation services across myriad healthcare settings. Its effectiveness in certain clinical contexts has led to the continued usage of telerehabilitation services despite the pandemic waning. The Society for Developmental and Behavioural Paediatrics has recently released a position statement on the promise of telehealth in delivering therapeutic interventions for children with neurodevelopmental disorders [49] and believes that this will also improve the equity of service provision for such children. Results from this trial will help inform providers and parents of children with ASD if telerehabilitation is a viable, legitimate alternative for early intervention, and contribute critically to the current gaps in randomised clinical trials in management and therapeutic interventions of ASD.

Regarding limitations, this trial is conducted as part of routine clinical care and operations. The CDU aims to provide interim support for children before they transition to the community-based early intervention programmes. As such, participants in the study will transit into the programmes once there is an available slot, and stop their intervention in CDU. This can happen at any time point throughout the intervention programme and will differ for each participant. However, most children will transit to community-based early intervention programmes only after completing the study as the waiting time for community-based early intervention programmes is about 1 year. In addition, a sensitivity analysis excluding participants who transit to community-based early intervention programmes before study completion may be conducted to examine its impact on trial results.

The outcomes of this trial are at best limited to a shorter period of observed child and parent benefits. The results of the trial will enhance our understanding of care delivery as part of a controlled clinical environment with interventions delivered by highly trained staff. Generalisation of our findings will be restricted to a replicable clinical environment with highly-supported technology access, in a country with high digital penetration like Singapore.

## Trial status

This study was approved by the institutional review board on 1 June 2018 (Protocol version 8, 19 August 2022). Recruitment started on 1 January 2019 and ended on 14 April 2022. All participants are anticipated to complete the study by 31 May 2023. The submission of this protocol for publication was delayed owing to unexpected changes in the study team composition and the previously emergent need to respond to the COVID-19 pandemic disruptions in the clinical service delivery.

## Supplementary Information

**Additional file 1.** Joint Engagement Rating Inventory itemsselected for this study.

**Additional file 2.** NDBI-Fi scale.

**Additional file 3.** Cost survey for participants in the standard arm.

**Additional file 4.** Cost survey for participants in the intervention arm.

**Additional file 5.** Parent satisfaction survey.

## Data Availability

The Principal Investigator will oversee the access to the final trial dataset. After the study statistician has analysed the final trial dataset, cleaned datasets will be made available to the study team for the purpose of the main publication. To ensure confidentiality, data dispersed to the study team members will not contain any participant identifiers. There are no contractual agreements that limit such access for any of the investigators. The Ministry of Health will receive a final report after the final dataset has been analysed. Any data required to support the protocol can be supplied on request.

## Abbreviations

ADOS: Autism Diagnostic Observation Schedule-2
ASD: Autism spectrum disorders
CDU: Child Development Unit
ELC: Early Learning Composite
FEIQoL: Families in Early Intervention Quality of Life
FSC: Foundational Skills Curriculum
JERI: Joint Engagement Rating Inventory
MSEL: Mullen’s Scale of Early Learning
PSI-SF: Parenting Stress Index-Short Form
VABS-III: Vineland Adaptive Behaviour Scales
