# Supplementary material for: Parent coaching via telerehabilitation for young children with autism spectrum disorder (ASD): study protocol for a randomised controlled trial
Source: Trials. 2023 Jul 19;24:462. doi: 10.1186/s13063-023-07488-6 (PMC10357872; doi:10.1186/s13063-023-07488-6)
Supplement: Supplementary file 1 — Additional file 1. Joint Engagement Rating Inventory itemsselected for this study. [file 13063_2023_7488_MOESM1_ESM.pdf]

File Name: Date: Rater Initials: _________

The Joint Engagement Rating Inventory (JERI)

Twenty-seven items are defined on the JERI master list, each with 7 points: 1 indicates a very low rating and 7 a very high rating.

Please note: For our study, only eight items are chosen for JERI. The number pertains to the specific item on the JERI master list.

Item	1=	2=	3=	4=	5=	6=	7=
Child Engagement State Items							
**22. Child's joint engagement**<br><br>Rating:	No episodes of the joint engagement state	Spends about or more than 11% of the scene in joint engagement, with majority being supported joint engagement	Spends about or more than 22% of the scene in joint engagement, with majority being supported joint engagement, with 1 or 2 occasions of coordinated joint engagement	Spends about or more than 33% of the scene in joint engagement, with majority being supported joint engagement with a few occasions of coordinated joint engagement	Spends about or more than 44% of the scene in joint engagement, with a mixture of supported joint engagement and coordinated joint engagement	Spends about or more than 55% of the scene in joint engagement, with a mixture of both supported and coordinated joint engagement, with slightly more occasions of coordinated joint engagement	Spends about or more than 66% of the scene in strikingly high quality coordinated joint engagement, of which most occasions are coordinated joint engagement rather than just supported joint engagement
Notes							

Video 1	Video 2	Video 3	Video 4
Joint Engagement duration: ___ %<br>Coordinated Joint Engagement occasions:	Joint Engagement duration: ___ %<br>Coordinated Joint Engagement occasions:	Joint Engagement duration: ___ %<br>Coordinated Joint Engagement occasions:	Joint Engagement duration: ___ %<br>Coordinated Joint Engagement occasions:

Child Activity Items							
**5. Child's initiation of communication**<br><br>**Rating:**	Child never makes a communicative initiation directed at caregiver, only directed at self	Child makes communicative initiations directed at caregiver on one or two occasions	Child makes communicative initiations directed at caregiver on three to four occasions	Child makes a mixture of communicative initiations directed at caregiver and self, with half of the communicative initiations directed at caregiver	Child makes a mixture of communicative initiations directed at caregiver and self, with slightly more communicative initiations directed at caregivers	Child makes a mixture of communicative initiations directed at caregiver and self, with more than 70% of communicative initiations directed at caregiver	Child continually makes communicative initiations directed at caregiver throughout the interaction

Notes

Video 1	Video 2	Video 3	Video 4

**6. Child's responsiveness to partner's communication**<br><br>Rating:	Child almost always resists or ignores caregiver's communication act	Child responds to or complies with caregiver's communicative act more than 11% of the time, with a few occasions of ignoring caregiver's communication act	Child responds to or complies with caregiver's communicative act more than 22% of the time, with a few occasions of resisting caregiver's communication act	Child responds to or complies with caregiver's communicative act more than 33% of the time. If the caregiver is passive and provides essentially no acts for the child to respond to, default to a code of 4.	Child responds to or complies with caregiver's communicative act more than 44% of the time	Child responds to or complies with caregiver's communicative act more than 55% of the time, with 1 or 2 occasions of eagerly anticipating caregiver's communicative act	Child responds to or complies with caregiver's communicative act more than 66% of the time and with few occasions of eagerly anticipating caregiver's communicative act

Notes

Video 1	Video 2	Video 3	Video 4

**7. Child's expressive language level and use**<br><br>**Rating:**	No expressive language	Uses 1 or 2 different single words	Uses about 5 different single words	Uses at least 10 different single words	Uses at least 3 different 2-word utterances	Uses at least 3 different 3-word utterances	Uses at least 3 different sentences

Notes

Video 1	Video 2	Video 3	Video 4

<u>Caregiver Items</u>

**10. Caregiver's scaffolding**<br><br>Rating:	Provides minimal support for the child's communication and/or actions on objects	Caregiver supports and extends the child's communication and/or actions little of the time, with evidence of modifying supports based on the child's ability or providing support well-timed relative to the child's current activity and child's response	Caregiver supports and extends the child's communication and/or actions little of the time, with evidence of modifying supports based on the child's ability and providing support well-timed relative to the child's current activity and child's response	Caregiver supports and extends the child's communication and/or actions some of the time, with evidence of modifying supports based on the child's ability or providing support well-timed relative to the child's current activity and child's response	Caregiver supports and extends the child's communication and/or actions some of the time, with evidence of modifying supports based on the child's ability and providing support well-timed relative to the child's current activity and child's response	Caregiver supports and extends the child's communication and/or actions most of the time, with clear evidence of modifying supports based on the child's ability or providing support well-timed relative to the child's current activity and child's response	Caregiver supports and extends the child's communication and/or actions most of the time, with clear evidence of modifying supports based on the child's ability and providing support well-timed relative to the child's current activity and child's response

Notes

Video 1	Video 2	Video 3	Video 4

**12. Caregiver's following in on child's focus**<br><br>Rating:	Caregiver rarely follows in on child's focus	Caregiver follows in on child's focus approximately 17% of the time	Caregiver follows in on child's focus approximately 33% of the time	Caregiver follows in on child's focus approximately 50% of the time	Caregiver follows in on child's focus approximately 67% of the time	Caregiver follows in on child's focus approximately 84% of the time	Caregiver almost continually joins and acts to sustain the child's interest

Notes

Video 1	Video 2	Video 3	Video 4

**13. Caregiver's affect**<br><br>Rating:	Caregiver is tense, disruptive, or affectively flat, to the point of being expressionless and subsequently very hard to read. Tension may be expressed by a strained voice and/or a tightened worried facial expression.	Caregiver is preoccupied, distracted, unresponsive, or bored.	Caregiver's affect appears flat.	Caregiver whose affect may be described as flat, with one or two occasions using one of 3 strategies - vocal inflections, facial expressions or body language.	Caregiver uses affect to enhance the communication by consistently using one of 3 strategies - vocal inflections, facial expressions or body language.	Caregiver uses affect to enhance the communication consistently using one of 3 strategies - vocal inflections, facial expressions or body language, with one or two occasions of using two or more strategies.	Caregiver uses affect to enhance the communication by consistently using two or more strategies - vocal inflections, facial expressions or body language throughout the interaction.

Notes

Video 1	Video 2	Video 3	Video 4

Dyadic Interaction Items							
**15. Sustainability of shared topic**<br><br>Rating:	No shared topic or fleeting topics throughout the video	Quickly attends to a topic and then moves on to the next topic for majority of the video	One or two sustained topics for at least 33% of the video	One or two sustained topics for half of the video	One or two sustained topics for at least 67% of the video	One or two sustained topics for at least 84% of the video	One or two sustained topics throughout the video

Notes

Video 1	Video 2	Video 3	Video 4
