# Supplementary material for: Parent coaching via telerehabilitation for young children with autism spectrum disorder (ASD): study protocol for a randomised controlled trial
Source: Trials. 2023 Jul 19;24:462. doi: 10.1186/s13063-023-07488-6 (PMC10357872; doi:10.1186/s13063-023-07488-6)
Supplement: Supplementary file 2 — Additional file 2. NDBI-Fi scale. [file 13063_2023_7488_MOESM2_ESM.pdf]

File Name: TH_______________ Timestamp: _________ - _________ Speech Level: Pre-verbal/Single words/Short phrases/Complex sentences

1. Face-to-face and on the child's level

1	2	3	4	5
The adult is rarely or never face-to-face and on the child's level. The adult is almost always standing, seated above the child, or behind the child.	The adult is occasionally face-to-face and on the child's level, however, most of the time, the adult may be standing, or seated above, behind; OR the adult is next to the child/kitty-corner for most of the session.	The adult is face-to-face and on the child's level for about half the session. Half the time, the adult may be standing, or seated above, behind, or next to the child.	The adult is usually face-to-face and on the child's level. When the child moves, the adult adjusts somewhat slowly, but eventually returns to a face-to-face position.	The adult is face-to-face and on the child's level throughout the session. When the child moves, the adult quickly adjusts position to return to a face-to-face position.

Timestamp

Face-to-face:

Not face-to-face:

2. Following the child's lead

1	2	3	4	5
The adult rarely or never joins the child in a child-led activity; OR signs of child interest or disinterest are largely ignored. Within home routines, the adult does not build in opportunities for the child to make choices. An adult who merely watches the child should be rated a 1.	The adult sometimes joins the child in a child-led activity, but most opportunities are missed; OR most signs of child's interest or disinterest are ignored. Within home routines, the adult usually does not build in opportunities for the child to make choices.	The adult joins in a child-led or child-chosen activity about half the time, but frequently directs the child to a certain activity, toy, or play action. Within home routines, opportunities for the child to choose are present but infrequent.	The adult joins in a child-led or child-chosen activity for the majority of the session, outside of direct teaching episodes. Most signs of child interest or disinterest are acknowledged. The adult may occasionally choose for the child or direct the child to play in new ways.	The adult almost always joins the child in a child-led activity, outside of direct teaching episodes. Signs of child interest or disinterest are acknowledged. In home routines, the adult creates several opportunities for child choice.

Notes

Was the parent-child dyad in the frame for at least 70% of the video?
Rater Initials:
Date:

3. Positive affect and animation

1	2	3	4	5
The adult's affect appears flat or uninterested throughout the session. The adult does not laugh, smile, or use exaggerated tone.	The adult occasionally displays exaggerated positive affect, but does not exaggerate vocal tone, gesture, and/or facial expression for the majority of the session; OR animation is poorly adjusted to the situation and child's sensory needs.	The adult uses a combination of vocal tone, gesture, and/or facial expression to display exaggerated positive affect for about half of the session, OR uses only one method for the majority of the session; OR animation is occasionally adjusted to the situation or child's sensory needs.	The adult usually uses a combination of vocal tone, gesture, and/or facial expression to display exaggerated positive affect, but misses several opportunities; OR the adult uses only one method of displaying positive affect throughout the session. The adult usually adjusts animation as needed.	The adult uses a combination of vocal tone, gesture, and/or facial expression to display exaggerated positive affect consistently throughout the session. The adult usually adjusts animation as needed.

Tick (√) if the adult uses the following

Vocal Quality or Tone	Gestures	Facial Expressions

4. Modeling appropriate language

1	2	3	4	5
The adult rarely or never models developmentally appropriate comments. All of the adult's comments may be far above or below the child's level, or the adult may exclusively ask rhetorical questions or give commands.	The adult occasionally models developmentally appropriate comments, but the majority of adult utterances are too far above or below the child's level, or most of the adult's utterances are questions or commands.	The adult models developmentally appropriate comments about half the time, but about half of utterances are too far above or below the child's level, or are questions or commands.	The adult models developmentally appropriate comments for most of the interaction, but some utterances are too far above or below the child's level, or the adult sometimes asks questions or gives commands.	The adult models developmentally appropriate comments throughout the interaction. No more than a few adult utterances are too far above or below the child's level, and there are no more than a few questions or commands.

Notes

5. Responding to attempts to communicate				
**1**	**2**	**3**	**4**	**5**
The adult rarely or never responds to the child's vocalizations and nonverbal attempts to communicate. The adult may make unrelated comments, or perform unrelated play acts in response. If the adult provides a few verbal responses but does not treat the child's communication as meaningful, rate a 1.	The adult occasionally provides meaningful responses to child's vocalizations and nonverbal attempts to communicate, but usually fails to respond, or usually responds un unrelated ways (i.e. low quality responses).	The adult sometimes responds to child's vocalizations and nonverbal attempts to communicate by clarifying or expanding on the child's utterances. About half the time, the adult fails to respond, or responds in unrelated ways. If the adult always repeats the child's utterances, but never expands on the child's communication, rate a 3.	The adult usually provides responses to the child's vocalizations and nonverbal attempts to communicate and treats them as meaningful, but occasionally fails to respond or misses some opportunities to clarify and expand the child's communication.	The adult nearly always responds to child's vocalizations and nonverbal attempts to communicate. This includes expanding or clarifying child utterances, and responding to the child's actions as meaningful. The adult misses no more than a few opportunities to respond.
Score **N/A** if the child does not vocalize or initiate communication with the adult.<br>*Notes*				

6. Using communicative temptations				
**1**	**2**	**3**	**4**	**5**
The adult never creates clear opportunities for the child to initiate.	The adult creates 1 clear opportunity for the child to communicate.	The adult creates 2 clear opportunities for the child to communicate.	The adult creates clear opportunities for the child to communicate 3-5 times.	The adult creates clear opportunities for the child to communicate more than 5 times.
Notes				

7. Frequency of direct teaching episodes (DTE)

1	2	3	4	5
The adult does not complete any DTE to teach skills.	The adult completes DTE 1 time in 5 minutes.	The adult completes DTE 2 times in 5 minutes.	The adult completes DTE 3-5 times in 5 minutes.	The adult completes DTE more than 5 times in 5 minutes.

	Adult's Instruction	Child's response/behaviour	Adult's response/reinforcement
DTO 1			
DTO 2			
DTO 3			
DTO 4			
DTO 5			
DTO 6			

8. Quality of direct teaching episodes

1	2	3	4	5
All DTE are of low quality (2 or fewer indicators). There are no high quality episodes.	2 quality indicators are consistently used across DTE. At least one high quality episode is present.	3 quality indicators are consistently used across DTE OR about half of episodes are poor quality (2 or fewer indicators).	4 quality indicators are present within most DTE. Few (if any) episodes are poor quality (2 or fewer indicators).	5 quality indicators are present within nearly all DTE. There are no poor quality episodes (2 or fewer indicators).

Score **N/A** if adult received a score of 1 on "7. Frequency of Direct Teaching"
Tick (√) if the adult uses the following

	Clear?	Develop. appropriate target?	Motivating and relevant?	Support correct response?	Natural and social reinforcement?
DTO 1					
DTO 2					
DTO 3					
DTO 4					
DTO 5					
DTO 6					
