# Supplementary material for: Parent coaching via telerehabilitation for young children with autism spectrum disorder (ASD): study protocol for a randomised controlled trial
Source: Trials. 2023 Jul 19;24:462. doi: 10.1186/s13063-023-07488-6 (PMC10357872; doi:10.1186/s13063-023-07488-6)
Supplement: Supplementary file 4 — Additional file 4. Cost survey for participants in the intervention arm. [file 13063_2023_7488_MOESM4_ESM.pdf]

Cost Survey (Telerehab)

Subject Number: ______________ **Date:** ______________

Transport

1) What mode of transport did you use to get here today?
   ☐ Taxi/Grab/Car hire
   ☐ Bus/MRT
   ☐ Private car
   ☐ Others: ____________________

2) How much did it cost?

   ____

3) Do you usually use this mode of transport to get to the clinic/CDU?
   ☐ Yes, move to next section
   ☐ No

4) If No, what is your usual mode of transport?
   ☐ Taxi/Grab/Car hire
   ☐ Bus/MRT
   ☐ Private car
   ☐ Others: ____________________

5) How much does it cost?

   ____

Employment

1) How many individuals in your household are formally employed?

   ____

2) Are you (Primary caregiver) currently formally employed?
   ☐ Yes
   - Full-time
   - Part-time

   ☐ No

3) How many hours do you currently work in a week?

   ____

4) What is your monthly income? (If not comfortable providing an exact value please give a range, for example if the monthly income is 3200 the range is 3000-4000)

   ____

5) [COLLECT ONLY AT BASELINE] Do you forsee the need to adjust your employment situation in light of this intervention?
☐ Yes
   - Reduce number of hours at work
   - Make arrangements to work remotely
   - Find a different job which allows me to spend more time with my child
   - Taking unpaid leave
   - Take time off
   - Others (please specify): _________________
☐ No

6) [COLLECT ONLY AT REVIEWS] Have there been any changes to your employment arrangement at home as a result of the intervention?
☐ Yes
   - Reduce number of hours at work
   - Make arrangements to work remotely
   - Find a different job which allows me to spend more time with my child
   - Taking unpaid leave
   - Take time off
   - Others (please specify): _________________
☐ No

7) Do you own a computer/laptop with capabilities for video calls such as skype or facetime?
☐ Yes
☐ No

Time Savings

1) How long did it take you to get to the clinic/CDU today? (In minutes)

____

2) Does it normally take you this long?
☐ Yes
☐ No
   - How long does it usually take? ____________

3) [COLLECT ONLY AT REVIEW] How long does it take for you to set up the computer before each telerehab session?

____

Care Costs

1) [COLLECT ONLY AT REVIEW] Did you pay for any form of assistance (Courses, caregivers, domestic helpers)?
☐ Yes
   - How much did you pay? ____________
☐ No

Engagement

1) In the past month, on average how much time (in hours) do you spend engaging (Speaking to, playing with, communicating with, doing activities) with your child per day?

____

2) [COLLECT ONLY AT REVIEW] In the past month, on average how much time (in hours) do you spend applying strategies learnt through the intervention with your child per day?

____

3) [COLLECT ONLY AT REVIEW] Has anyone else in your household been taught to apply these strategies when engaging with your child? Please tick all that apply
☐ Domestic Helper
☐ Other parent
☐ Siblings
☐ Grandparent(s)
☐ Other relatives (Eg. Uncles, aunties and cousins)
☐ Friends
☐ Others (Please Specify): __________________
