# Supplementary material for: Parent coaching via telerehabilitation for young children with autism spectrum disorder (ASD): study protocol for a randomised controlled trial
Source: Trials. 2023 Jul 19;24:462. doi: 10.1186/s13063-023-07488-6 (PMC10357872; doi:10.1186/s13063-023-07488-6)
Supplement: Supplementary file 5 — Additional file 5. Parent satisfaction survey. [file 13063_2023_7488_MOESM5_ESM.pdf]

Telerehab Program Parent Satisfaction Survey

Subject Number: ______________ **Date:** ______________

Please rate your response to the following statements on a scale between 1 and 5. 1 being strongly disagree and 5 being strongly agree.

	Strongly Agree	Agree	Neutral	Disagree	Strongly Disagree
1. The intervention program has helped my child's learning					
2. The telerehab program meets my child's needs					
3. Telerehab program intervention is simple to use and easy to understand					
4. The information shared has been clear and useful to me					
5. I am able to use routines and activities introduced in the telerehab program regularly to help my child's learning					
6. I am confident in my ability to help my child learn through applying the strategies I learnt in the Telerehab program to help my child's development					
7. I understand the role of activities and daily routines in helping my child learn					

8. I would recommend the Telerehab program to other parents					
9. The frequency of intervention was sufficient in helping me to be competent and confident in helping my child learn.					
10. Support and resources provided by the therapists was adequate in helping me learn to support my child					
11. I did not experience any difficulties during the tele-conference with the therapists weekly					
12. Tele-conferencing with therapists helped me save time as compared to going down to the clinic					
13. The home environment has been conducive for the telerehab program					
